# The α-helix-facilitating amino acid sequence EAAAK dramatically increases the antiviral activity of SARS-CoV-2 fusion-inhibitory peptides via the endosome fusion pathway

**DOI:** 10.1128/jvi.01067-26

**Published:** 2026-09-08

**Authors:** Zhixin Yao, Yuanmei Zhu, Nian Liu, Yue Hu, Yuxian He

**Affiliations:** 1 Key Laboratory of Pathogen Infection Prevention and Control (Ministry of Education), State Key Laboratory of Respiratory Health and Multimorbidity, National Institute of Pathogen Biology, Chinese Academy of Medical Sciences and Peking Union Medical College 12501, Beijing, People's Republic of China; 2 NHC Key Laboratory of Systems Biology of Pathogens, National Institute of Pathogen Biology, Chinese Academy of Medical Sciences and Peking Union Medical College 12501, Beijing, People's Republic of China; University of North Carolina at Chapel Hill, Chapel Hill, North Carolina, USA

**Keywords:** SARS-CoV-2, membrane fusion, fusion inhibitor, peptide, EAAAK linker

## Abstract

The SARS-CoV-2 fusion-inhibitory lipopeptide IPB29 features a unique design incorporating the α-helix-promoting EAAAK sequence as a rigid linker between the peptide backbone and the conjugated lipid, exhibiting greatly improved antiviral activity compared to lipopeptides with conventional flexible linkers. Here, we characterized its mechanism of action and uncovered several critical insights. First, IPB29 remained the most potent inhibitor of emerging Omicron subvariants compared to IPB24 and IPB28, which share the same peptide sequence but employ flexible polyethylene glycol or glycine-serine repeat linker. Notably, this enhanced potency was abolished in Vero E6 cells stably expressing TMPRSS2, where viral entry occurs via the plasma membrane fusion pathway. Visualization using FITC-labeled lipopeptides demonstrated that IPB29 binds to and enters target cells more efficiently. Subsequently, we generated two unconjugated template peptides, P43 (containing EAAAK) and P37 (lacking EAAAK). P43 exhibited superior inhibitory activity compared to P37 in Huh-7 and 293T/ACE2 cells, but not in Vero E6-TMPRSS2 cells. Both peptides showed comparable efficacy in blocking S protein-driven cell-cell fusion. Although P43 displayed enhanced α-helicity relative to P37, both peptides exhibited similar target-binding affinities. Instead, the cellular uptake of P43 was markedly more efficient, further supporting that the improved antiviral effect is attributable to the inhibition of the viral endosomal fusion pathway. Collectively, our studies provide molecular insights into the antiviral mechanism of IPB29 and propose the short EAAAK sequence as a generalizable strategy for enhancing the efficacy of peptide-based therapeutics.

**IMPORTANCE:**

The development of effective therapeutics against SARS-CoV-2 remains an urgent priority. IPB29, a potent broad-spectrum lipopeptide fusion inhibitor currently in clinical trials, requires mechanistic elucidation. Here, we demonstrate that the C-terminal EAAAK motif in IPB29—originally designed as a rigid linker connecting the peptide backbone to the lipid moiety—is critical for its antiviral activity and functions via the endosomal fusion pathway. These findings reveal a generalizable strategy for optimizing antiviral drug design.

## INTRODUCTION

The infection of cells by SARS-CoV-2 requires fusion between viral and cellular membranes, a process initiated by the viral trimeric spike (S) glycoprotein (1–3). The S protein is composed of a surface S1 subunit and a transmembrane S2 subunit. The S1 subunit contains a receptor-binding domain (RBD) that engages the host cell receptor, human angiotensin-converting enzyme 2 (ACE2). This binding triggers conformational changes, promotes hydrolysis of the S1/S2 complex, and activates the fusion machinery within the S2 subunit. Subsequently, the fusion peptide (FP) at the N-terminus of S2 is exposed and inserts into the target cell membrane, forming a pre-hairpin intermediate that bridges the viral and cellular membranes. The heptad repeat-2 (HR2) region of S2 then aligns antiparallelly with the hydrophobic grooves of the trimeric heptad repeat-1 (HR1) coiled-coil, leading to the formation of a stable six-helix bundle (6-HB). This structure pulls the viral and cellular membranes into close proximity, ultimately driving membrane fusion and viral entry. Given its central role in the early stages of infection, the S protein represents a key target for antiviral drug and vaccine development (4, 5).

Although the acute phase of the COVID-19 pandemic has subsided, the continuous evolution of SARS-CoV-2 has led to the emergence of numerous variants of concern (VOCs). These variants often develop resistance to existing antiviral drugs and vaccines, underscoring the persistent threat of the virus and highlighting the critical need to develop broad-spectrum antiviral agents with distinct mechanisms of action. Such agents are essential for refining therapeutic strategies to keep pace with viral evolution (6–8). Notably, while the S1 subunit undergoes rapid mutation, the amino acid sequences of the S2 subunit remain highly conserved, even among divergent VOCs such as Omicron. This stability makes the S2 subunit an attractive target for intervention. Indeed, peptide-based membrane fusion inhibitors—typically derived from the HR2 or HR1 regions—consistently demonstrate high potency and broad-spectrum antiviral activity, not only against SARS-CoV-2 variants but also against other human coronaviruses (9–11). Mechanistically, these inhibitors act by competitively binding to the counterpart HR1 or HR2 domain, thereby blocking the formation of the 6-HB structure required for membrane fusion.

**Fig 1:**
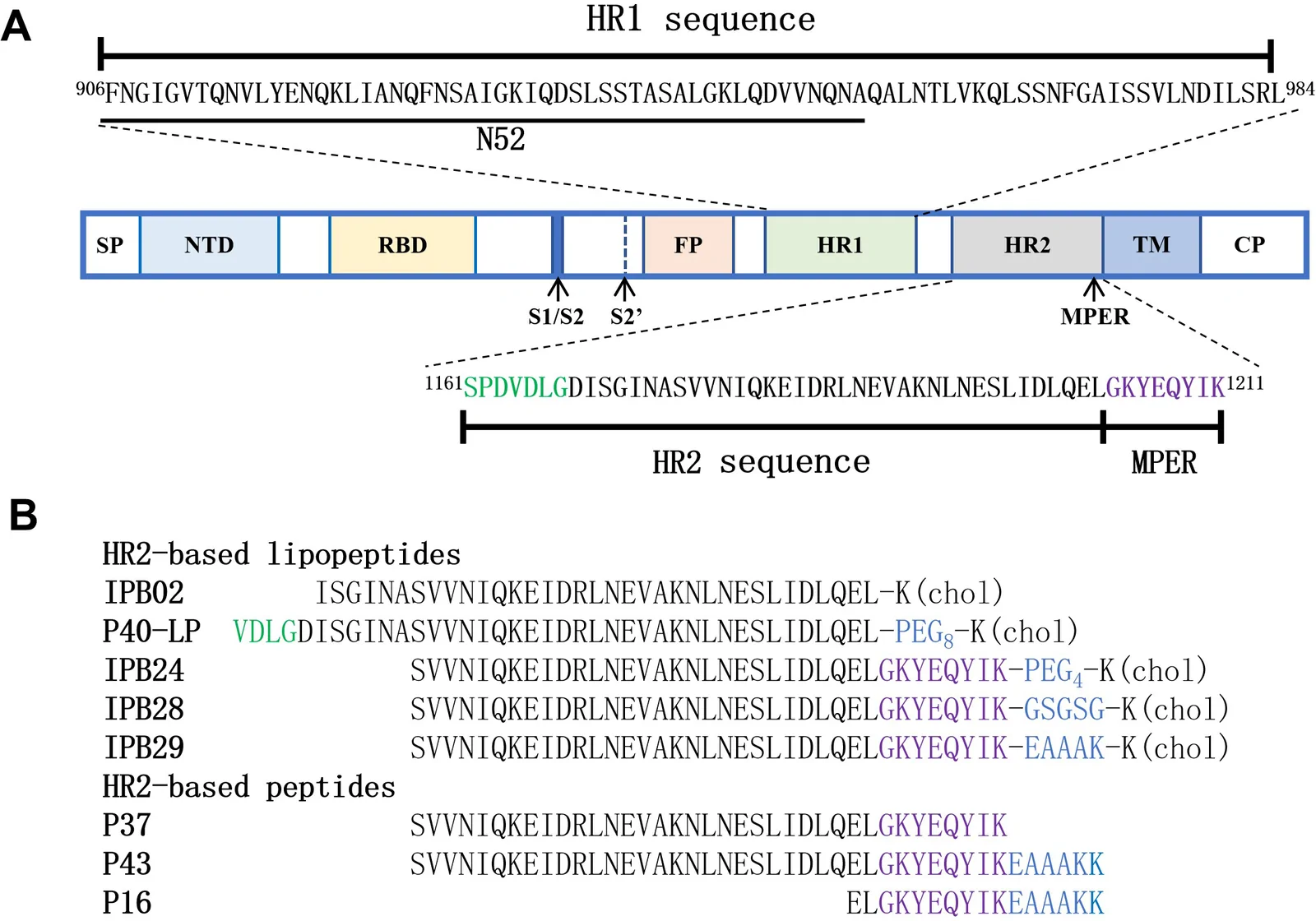
SARS-CoV-2 spike protein and HR2-derived fusion inhibitors. (**A**) Schematic representation of the spike protein and sequences of the heptad repeat 1 (HR1) and heptad repeat 2 (HR2) regions. Abbreviations: SP, signal peptide; NTD, N-terminal domain; RBD, receptor-binding domain; FP, fusion peptide; HR1, heptad repeat 1; HR2, heptad repeat 2; MPER, membrane-proximal external region; TM, transmembrane domain; CP, cytoplasmic peptide. The S1/S2 and S2′ cleavage sites are indicated with arrows. The HR1 and HR2 sequences are shown, with the HR1-derived peptide N52 underlined. (**B**) Design of SARS-CoV-2 fusion inhibitors based on the HR2 sequence. N-terminal extensions are highlighted in green, and C-terminal extensions are highlighted in purple. Chol, cholesterol; PEG_8_/PEG_4_, 8- or 4-unit polyethylene glycol linker.

Since the initial COVID-19 outbreak, we have been dedicated to developing SARS-CoV-2 fusion inhibitors, generating a series of peptides and lipopeptides with potent antiviral activity (12–16). In general, unconjugated peptide-based fusion inhibitors exhibit significantly weaker activity compared to their lipid-conjugated counterparts, a trend also observed in our prior research on HIV fusion inhibitors (17, 18). This holds true for SARS-CoV-2 as well, as demonstrated by the EK1 peptide and its lipid derivative EK1C4 (19), the HR2-P40 peptide and its lipid derivative P40-LP (15), and the IPB19 peptide and its lipid derivatives IPB24 and IPB29 (13, 16). In designing lipopeptide-based fusion inhibitors, the linker between the peptide and lipid moieties is critical for antiviral potency. Notably, while previously reported lipopeptide fusion inhibitors exclusively employed flexible linkers—typically short polyethylene glycol (PEG_4_ or PEG_8_) or glycine-serine repeats (GSGSG) or a combination thereof—IPB29 was uniquely engineered with the α-helix-promoting amino acid sequence “EAAAK” as a rigid linker, as depicted in Fig. 1. Compared to inhibitors with the same peptide sequence but a flexible linker, IPB29 unexpectedly demonstrated markedly enhanced activity against diverse SARS-CoV-2 variants (9, 16). In animal models, IPB29 showed high therapeutic and prophylactic efficacy. Although IPB29 has already advanced to a phase III clinical trial, the functional mechanism underlying the “EAAAK” sequence is not fully clear (20). In this study, we focused on characterizing fusion inhibitors containing the “EAAAK” motif and obtained key findings, as detailed in the following Results section.

## RESULTS

### IPB29 exhibits potent inhibition against emerging Omicron subvariants

**Fig 2:**
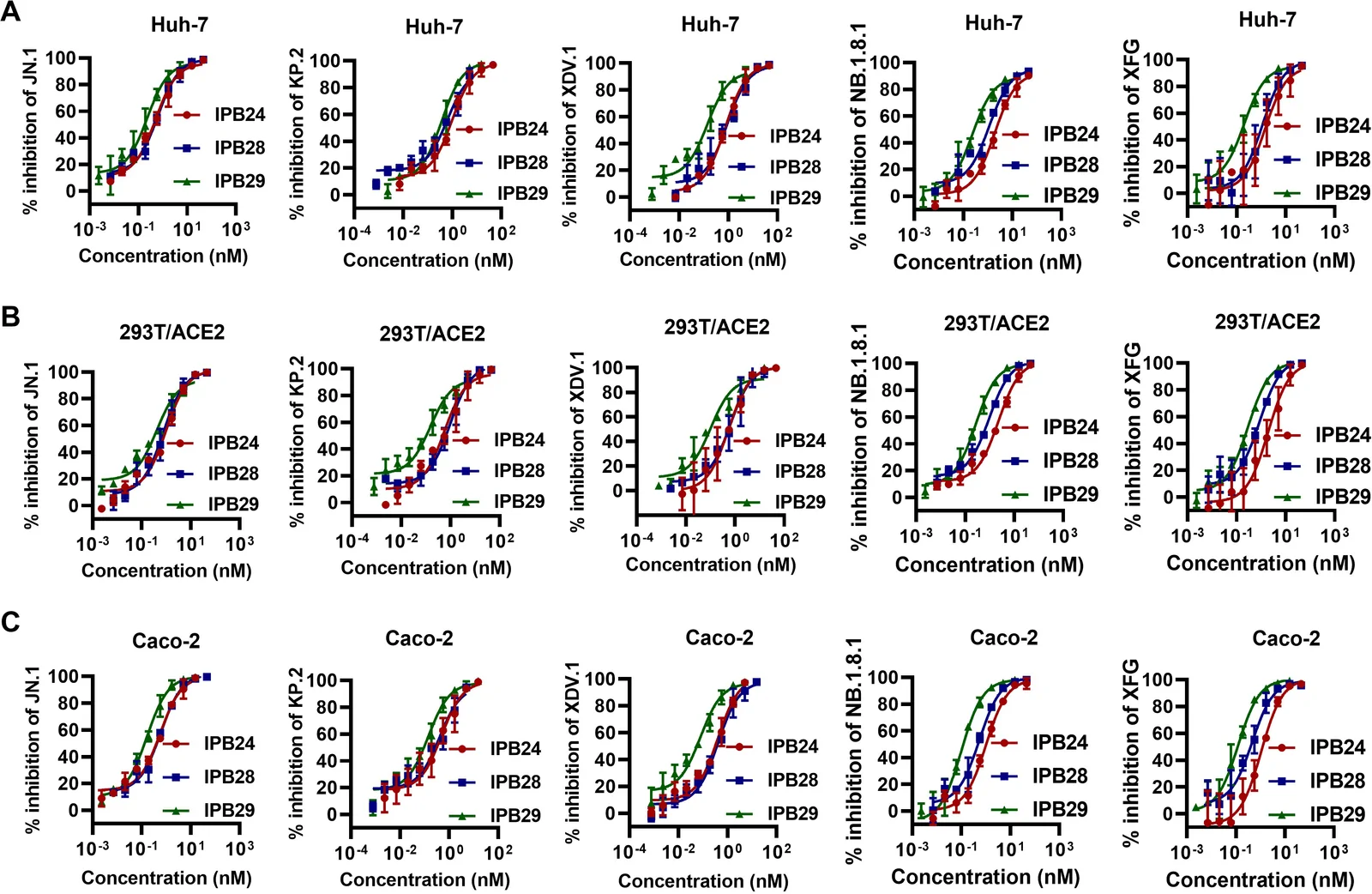
Inhibition of emerging Omicron variants by lipopeptide-based fusion inhibitors. Inhibitory activities of IPB24, IPB28, and IPB29 against the Omicron subvariants JN.1, KP.2, XDV.1, NB.1.8.1, and XGF were assessed in Huh-7 (**A**), 293T/ACE2 (**B**), and Caco-2 (**C**) cells using a pseudovirus-based single-cycle infection assay. Data represent mean ± SD of three independent experiments, each performed in triplicate.

We previously demonstrated that IPB29 possesses significantly enhanced anti-coronavirus activity compared to IPB24 and IPB28, which share an identical peptide backbone but incorporate a flexible PEG_4_ or GSGSG linker, respectively (Fig. 1). Here, we extended this comparison to newly emerged Omicron subvariants, including JN.1, KP.2, XDV.1, NB.1.8.1, and XFG. Single-cycle infection assays using pseudoviruses bearing the S proteins of these variants revealed that IPB29 consistently exhibited superior inhibitory potency across all tested cell lines (Fig. 2, Table 1). Specifically, in Huh-7 cells, IPB29 inhibited JN.1, KP.2, XDV.1, NB.1.8.1, and XFG with IC_50_ values of 0.23, 0.4, 0.14, 0.25, and 0.2 nM, respectively—outperforming IPB24 (0.52, 0.97, 0.78, 2, and 1.56 nM) and IPB28 (0.64, 0.67, 0.93, 1.15, and 1.14 nM). Similarly, in 293T/ACE2 cells, IPB29 achieved IC_50_ values of 0.44 nM (JN.1), 0.2 nM (KP.2), 0.12 nM (XDV.1), 0.27 nM (NB.1.8.1), and 0.3 nM (XFG), while in Caco-2 cells, the corresponding values were 0.17, 0.15, 0.09, 0.1, and 0.12 nM. Overall, IPB29 was approximately 2- to 11-fold more potent than IPB24 and IPB28 against all tested variants across the three cell lines.

**TABLE 1:** Inhibitory activities of lipopeptide and peptide-based fusion inhibitors against emerging Omicron subvariants^*a*^

SARS-CoV-2	Target cells	JN.1		KP.2		XDV.1		NB.1.8.1		XFG	
		IC_50_ (nM)	*P* value	IC_50_ (nM)	*P* value	IC_50_ (nM)	*P* value	IC_50_ (nM)	*P* value	IC_50_ (nM)	*P* value
Lipopeptides											
IPB24	Huh-7	0.52 ± 0.09	0.0097	0.97 ± 0.19	0.0002	0.78 ± 0.17	<0.0001	2.00 ± 0.82	<0.0001	1.56 ± 0.74	0.0002
IPB28	Huh-7	0.64 ± 0.10	0.0004	0.67 ± 0.30	0.1559	0.93 ± 0.08	<0.0001	1.15 ± 0.23	<0.0001	1.14 ± 0.33	<0.0001
IPB29	Huh-7	0.23 ± 0.07	–	0.40 ± 0.11	–	0.14 ± 0.01	–	0.25 ± 0.14	–	0.20 ± 0.02	–
IPB24	293T/ACE2	0.99 ± 0.16	0.0086	0.80 ± 0.37	0.0036	0.57 ± 0.08	0.0012	2.29 ± 0.05	<0.0001	1.98 ± 0.71	<0.0001
IPB28	293T/ACE2	0.84 ± 0.53	0.0784	1.03 ± 0.40	<0.0001	0.90 ± 0.78	<0.0001	0.97 ± 0.15	<0.0001	0.89 ± 0.17	<0.0001
IPB29	293T/ACE2	0.44 ± 0.08	–	0.20 ± 0.11	–	0.12 ± 0.06	–	0.27 ± 0.05	–	0.30 ± 0.04	–
IPB24	Caco-2	0.59 ± 0.15	<0.0001	0.55 ± 0.23	0.0025	0.41 ± 0.09	<0.0001	1.06 ± 0.28	<0.0001	1.15 ± 0.29	<0.0001
IPB28	Caco-2	0.60 ± 0.05	<0.0001	0.46 ± 0.17	0.0198	0.47 ± 0.11	<0.0001	0.54 ± 0.10	<0.0001	0.47 ± 0.11	<0.0001
IPB29	Caco-2	0.17 ± 0.03	–	0.15 ± 0.02	–	0.09 ± 0.03	–	0.10 ± 0.02	–	0.12 ± 0.02	–
Peptides											
P37	Huh-7	3,774.33 ± 1,120.98	0.0003	3,906.00 ± 1,067.35	<0.0001	5,178.67 ± 1,659.17	<0.0001	6,800.00 ± 3,499.73	<0.0001	4,635.00 ± 1,552.56	0.0002
P43	Huh-7	641.13 ± 157.05	–	461.23 ± 118.57	–	596.37 ± 173.61	–	679.77 ± 259.03	–	561.23 ± 225.30	–
P37	293T/ACE2	8,105.33 ± 3,333.14	<0.0001	8,676.33 ± 3,518.11	<0.0001	6,501.67 ± 1,446.28	0.0035	12,103.00 ± 1,081.48	<0.0001	8,163.33 ± 2,990.55	<0.0001
P43	293T/ACE2	379.43 ± 20.16	–	481.43 ± 159.59	–	1,418.33 ± 362.44	–	1,052.33 ± 802.06	–	470.07 ± 208.68	–

^*a*^ Data represent mean ± SD of three independent experiments, each performed in triplicate. The statistical significance of the differences in logIC_50_ between groups was determined using the extra sum-of-squares *F* test: ns, not significant (*P* > 0.05); *P < 0.05; **P < 0.01; ***P < 0.001; ****P < 0.0001; –, not applicable for statistical analysis.

### The enhanced antiviral activity of IPB29 depends on the viral endosome fusion pathway

**Fig 3:**
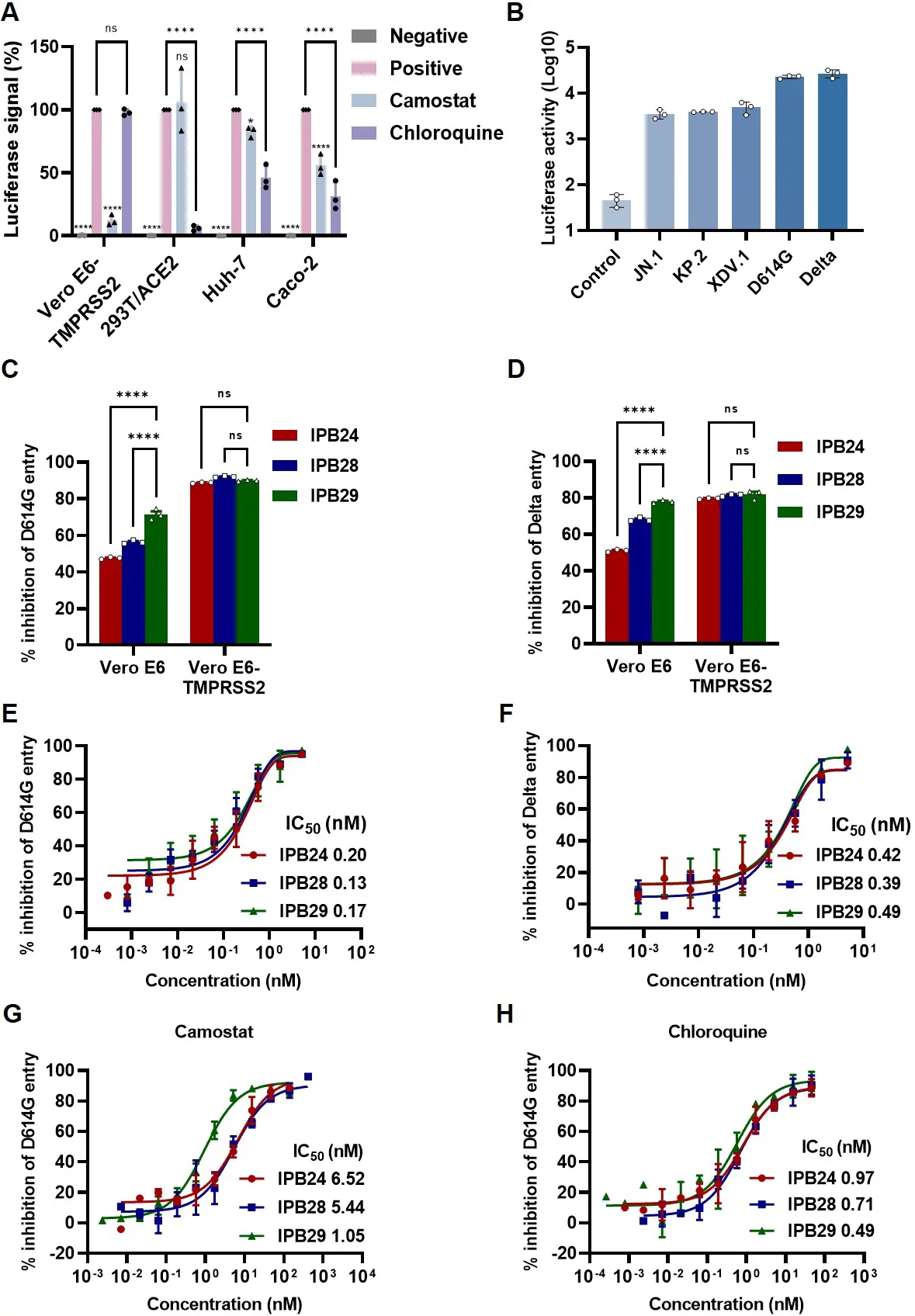
Antiviral activities of lipopeptide-based fusion inhibitors correlate with SARS-CoV-2 entry pathways. (**A**) Infectivity of D614G pseudovirus in Vero E6-TMPRSS2, 293T/ACE2, Huh-7, and Caco-2 cells treated with DMEM (positive control) or pathway-specific chemical inhibitors (Camostat at 20 μM or Chloroquine at 1 μM) for 1 h. Uninfected cells served as a negative control. Luciferase signals were normalized to the positive control (set as 100%). (**B**) Infectivity of SARS-CoV-2 variants (D614G, Delta, JN.1, KP.2, and XDV.1) in Vero E6-TMPRSS2 cells. (**C and D**) Inhibitory activities of lipopeptides against D614G (**C**) and Delta (**D**) in Vero E6 cells (5 nM) and Vero E6-TMPRSS2 cells (1 nM). (**E and F**) Inhibitory activities of lipopeptides against D614G (**E**) and Delta (**F**) in Vero E6-TMPRSS2 cells. (**G and H**) Inhibitory activities of lipopeptides against D614G in Huh-7 cells pretreated with 20 μM of Camostat (**G**) or 1 μM of Chloroquine (**H**) for 1 h. Data represent mean ± SD from three independent experiments, each performed in triplicate. Statistical significance in panels A, C, and D was determined by two-way analysis of variance followed by Dunnett’s test: ns, not significant (*P* > 0.05); *P < 0.05; **P < 0.01; ***P < 0.001; ****P < 0.0001.

SARS-CoV-2 enters host cells via two principal pathways: endosome-mediated membrane fusion and TMPRSS2-dependent plasma membrane fusion. Given that lipopeptide-based fusion inhibitors can bind to and enter target cells, we sought to determine whether their inhibitory efficacy correlates with the viral entry route. Consistent with previous reports, SARS-CoV-2 (D614G) infected 293T/ACE2 cells primarily via endocytosis, infected Huh-7 and Caco-2 cells through both endocytosis and plasma membrane fusion, and predominantly utilized the plasma membrane fusion pathway in Vero E6 cells stably expressing TMPRSS2 (Vero E6-TMPRSS2; Fig. 3A). We initially attempted to evaluate the inhibitory activities of IPB24, IPB28, and IPB29 against the JN.1, KP.2, and XDV.1 pseudoviruses in Vero E6-TMPRSS2 cells. However, unlike the D614G and Delta variants—which efficiently utilize TMPRSS2—these newer Omicron subvariants infected Vero E6-TMPRSS2 cells poorly (Fig. 3B), aligning with prior observations that mutations in the Omicron spike protein impair entry in cells with high cell-surface TMPRSS2 expression (21–23). Consequently, D614G and Delta pseudoviruses were used for subsequent analyses.

To specifically assess the impact of TMPRSS2 expression, we compared the inhibitory activities of the lipopeptides in Vero E6 cells (which lack TMPRSS2 and rely exclusively on endosomal entry) and Vero E6-TMPRSS2 cells. As shown in Fig. 3C and D, IPB29 remained the most potent inhibitor of D614G and Delta in Vero E6 cells. In contrast, all three lipopeptides exhibited comparable inhibitory activities in Vero E6-TMPRSS2 cells, with IC_50_ values of 0.20, 0.13, and 0.17 nM against D614G, and 0.42, 0.39, and 0.49 nM against Delta for IPB24, IPB28, and IPB29, respectively (Fig. 3E and F). Furthermore, we compared their inhibitory activities against D614G in Huh-7 cells pretreated with camostat (to block plasma membrane fusion) or chloroquine (to block endosomal fusion). In camostat-treated cells, IPB29 was over fivefold more potent than IPB24 and IPB28; however, this difference was reduced to less than twofold in chloroquine-treated cells (Fig. 3G and H). These results demonstrate that the enhanced antiviral activity of IPB29 is critically dependent on the endosomal fusion pathway.

### IPB29 efficiently binds to the cell membrane and enters the cells

**Fig 4:**
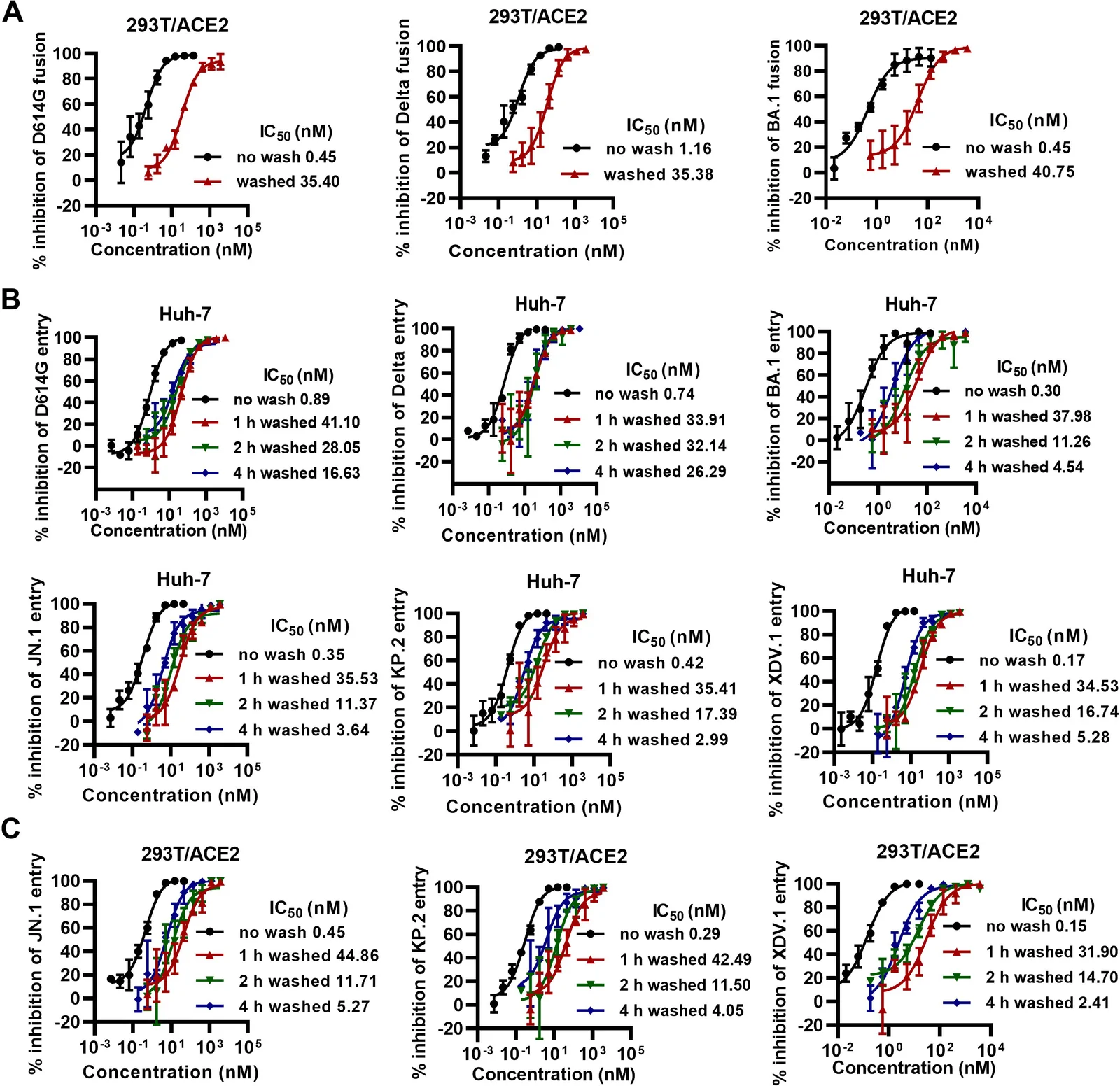
Activity of cell-associated IPB29 against SARS-CoV-2 variants. (**A**) Sustained inhibitory activity of cell-bound IPB29 against spike protein-mediated cell–cell fusion. After pre-incubation with 293T/ACE2 cells for 1 h and thorough washing, the remaining inhibitory activity of IPB29 against D614G, Delta, and BA.1 variants was measured using a dual DSP-based cell fusion assay. (**B and C**) Sustained antiviral activity of cell-associated IPB29 against SARS-CoV-2 pseudoviruses. IPB29 was pre-incubated with Huh-7 (**B**) or 293T/ACE2 (**C**) cells for 1, 2, or 4 h, washed thoroughly, and its residual inhibitory activity against multiple SARS-CoV-2 variants was assessed using a pseudovirus-based single-cycle infection assay. Data are presented as mean ± SD from three independent experiments, each performed in triplicate.

It is hypothesized that lipopeptide-based viral fusion inhibitors can bind to and enter target cells, thereby increasing their local concentration at the site of membrane fusion. To investigate this for IPB29, we employed several experimental approaches. First, we evaluated the membrane-binding ability of IPB29 using a DSP-based cell-cell fusion assay. IPB29 was pre-incubated with 293T/ACE2 cells; after extensive washing to remove unbound inhibitor, the residual inhibitory activity of cell-bound IPB29 against S protein-mediated cell fusion (induced by D614G, Delta, and BA.1 spikes) was measured. As shown in Fig. 4A, IPB29 retained substantial inhibitory activity even after washing, confirming its capacity to stably associate with the cell membrane. Second, we characterized IPB29 using a pseudovirus-based single-cycle infection assay, which reflects both membrane binding and potential cellular internalization. Following pre-incubation with Huh-7 cells for varying durations, IPB29 was washed out, and its residual antiviral activity was determined. IPB29 maintained potent inhibition against all tested pseudoviruses post-wash (Fig. 4B). Notably, its inhibitory potency increased with longer pre-incubation periods, particularly against the Omicron variants, which preferentially utilize the endocytic entry pathway. This time-dependent enhancement of cell-associated IPB29 activity was further confirmed against three newer Omicron subvariants in 293T/ACE2 cells (Fig. 4C). To verify the efficiency and specificity of the washing procedure, we included control entry inhibitors—Camostat, the fusion-inhibitory peptide P39, and the RBD-directed fusion protein LCB1T-Fc (15, 24). In contrast to IPB29, the inhibitory activities of these compounds were completely abolished following washing (Fig. S1A).

**Fig 5:**
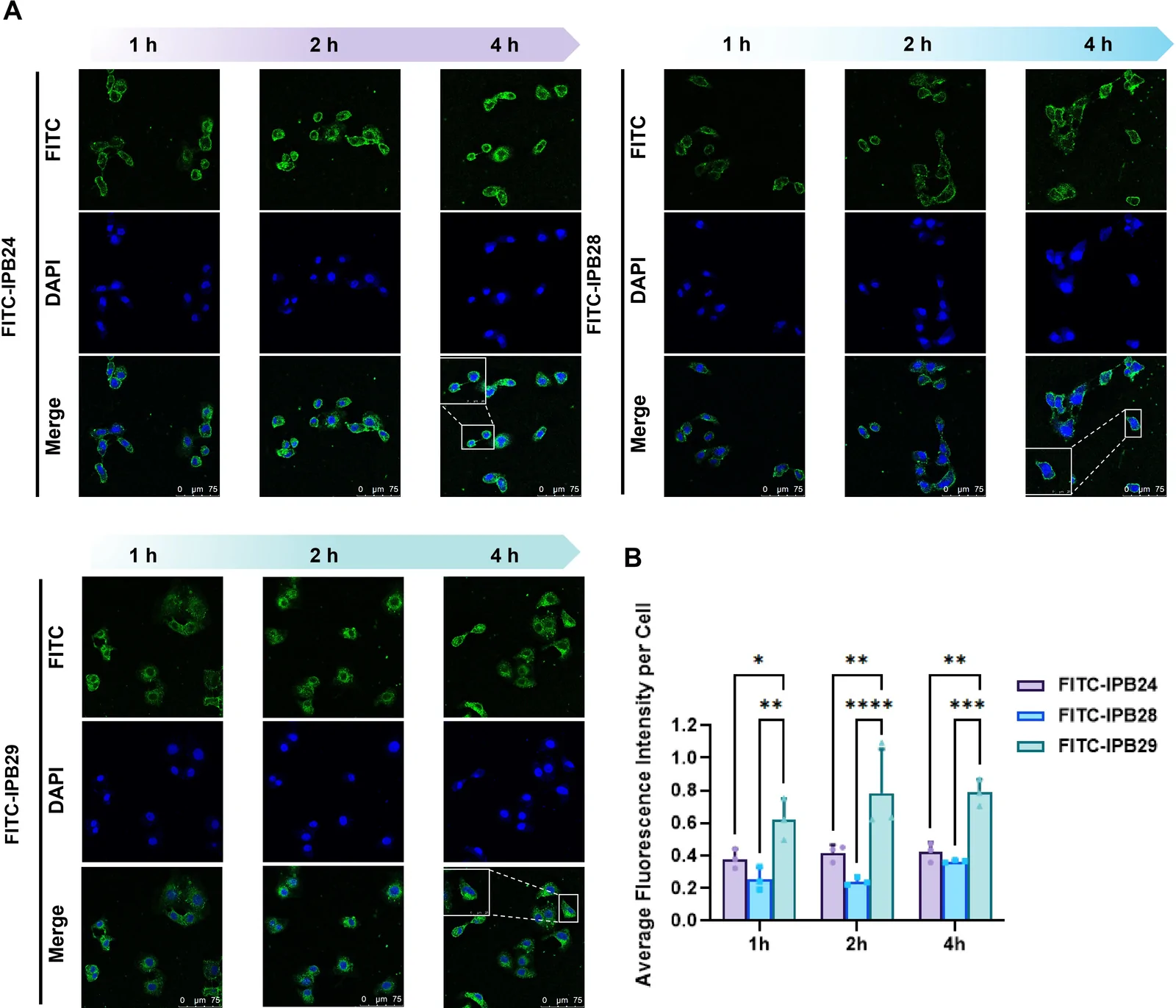
Cellular localization of lipopeptides. (**A**) Cellular internalization of FITC-labeled lipopeptides. Vero E6 cells were incubated with 25 µM of FITC-IPB24, FITC-IPB28, or FITC-IPB29 for 30 min, 1 h, 2 h, and 4 h. Cells were then fixed, counterstained with DAPI, washed, and imaged by confocal microscopy. Images are representative of two independent experiments with similar results. In each independent experiment, images were acquired from three random fields. (**B**) Per-cell fluorescence intensity was measured per condition per field of view using Fiji (ImageJ), and error bars indicate SEM (*n* = 3). Statistical analysis was performed using two-way analysis of variance followed by Dunnett’s test: ns, not significant (*P* > 0.05); *P < 0.05; **P < 0.01; ***P < 0.001; ****P < 0.0001.

To directly visualize membrane binding and cellular uptake, we conducted confocal microscopy analyses using N-terminally fluorescein isothiocyanate (FITC)-labeled lipopeptides. Vero E6 cells were incubated with increasing concentrations (0, 5, 10, or 25 μM) of FITC-IPB24, FITC-IPB28, or FITC-IPB29 for 1 h. Confocal imaging revealed concentration-dependent binding of all three lipopeptides to the cell membrane (Fig. S1B). Time-course analysis at 25 μM further highlighted kinetic differences among them (Fig. 5). FITC-IPB29 achieved markedly higher cytoplasmic accumulation and penetrated more deeply into the perinuclear region within 1–2 h. By 4 h, FITC-IPB29 produced the most intense and widespread cytoplasmic fluorescence, whereas FITC-IPB24 and FITC-IPB28 yielded comparatively weaker signals. Notably, nuclear colocalization was observed predominantly in the FITC-IPB29-treated group. Collectively, these findings demonstrate that IPB29 exhibits superior cellular entry kinetics compared with IPB24 and IPB28 and establish a direct link between its efficient intracellular uptake and its potent antiviral activity against viruses utilizing the endocytic membrane fusion pathway.

### The EAAAK sequence dramatically increases the antiviral activity of template peptides

**Fig 6:**
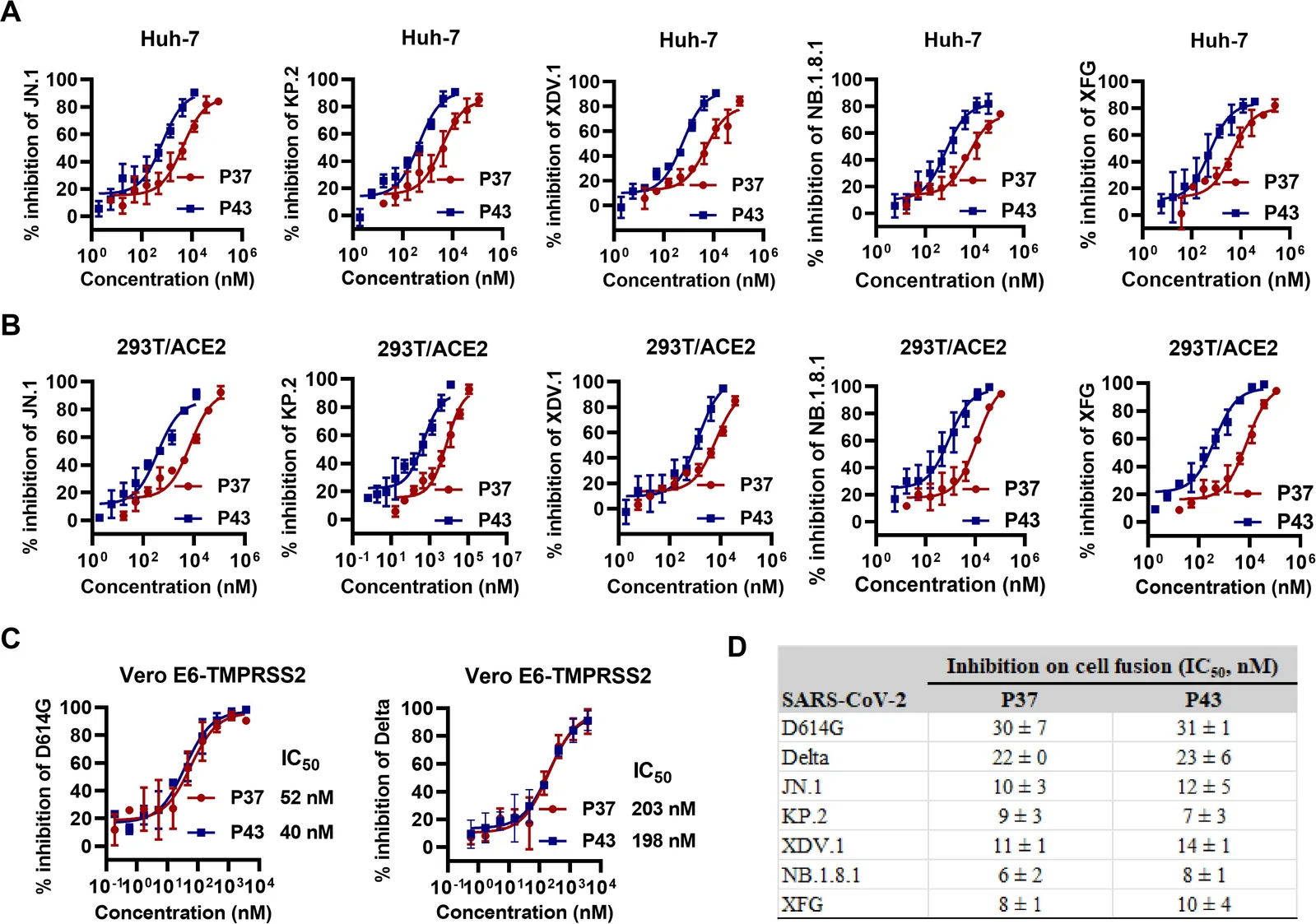
Inhibition of SARS-CoV-2 variants by P37 and P43. (**A–C**) Inhibitory activities of P37 and P43 against diverse SARS-CoV-2 variants were evaluated in Huh-7 (**A**), 293T/ACE2 (**B**), and Vero E6-TMPRSS2 (**C**) cells using a pseudovirus-based single-cycle infection assay. (**D**) Inhibitory activities of P37 and P43 against spike-mediated cell fusion determined by a DSP-based cell fusion assay. Data are presented as mean ± SD from three independent experiments, each performed in triplicate.

Our previous data highlighted the importance of the EAAAK sequence in IPB29 as a rigid linker, which enhanced helical stability, membrane binding, and cellular entry. To further investigate whether this sequence contributes directly to antiviral activity—rather than merely serving as a conjugation linker—we synthesized two unconjugated template peptides: P43 (containing EAAAK) and P37 (without EAAAK), as illustrated in Fig. 1. Using a pseudovirus-based single-cycle infection assay, we found that P43 exhibited significantly stronger inhibition than P37 against JN.1, KP.2, XDV.1, NB.1.8.1, and XFG pseudoviruses (Fig. 6A and B, Table 1). Specifically, in 293T/ACE2 cells, P43 was 21-fold more potent against JN.1 and 18-fold more potent against KP.2 than P37. Similarly, in Huh-7 cells, P43 exhibited over an 8- and 10-fold increase in potency against XDV.1 and NB.1.8.1, respectively. In contrast, both peptides displayed comparable potency against the D614G and Delta variants in Vero E6-TMPRSS2 cells (Fig. 6C). Furthermore, a DSP-based cell-cell fusion assay revealed that P43 and P37 possessed similar IC₅₀ values in blocking spike-driven membrane fusion (Fig. 6D)—a process independent of the endocytic entry pathway. Taken together, these results demonstrated that the EAAAK sequence selectively enhances antiviral potency via the endosomal fusion pathway, an effect that is particularly pronounced in cholesterol-free peptide fusion inhibitors.

### The EAAAK sequence enhances the α-helicity of P43 without altering target binding affinity

**Fig 7:**
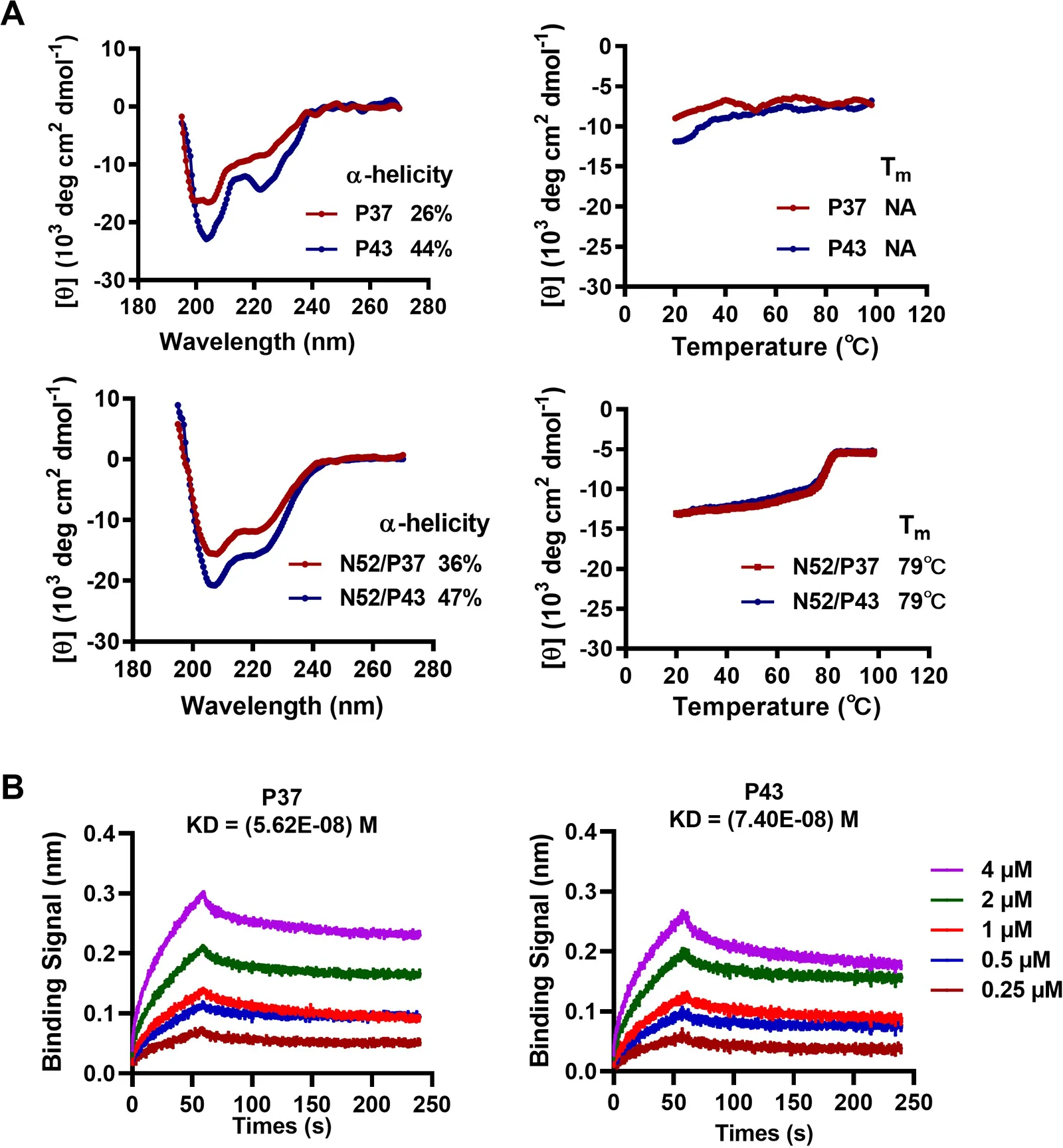
Secondary structure and target binding of P37 and P43 peptides. (**A**) Circular dichroism (CD) spectroscopy analysis of α-helical content and thermal stability of P37 and P43 alone or in complex with the HR1-derived peptide N52 (peptide concentration: 10 μM). (**B**) Binding kinetics of P37 and P43 to biotinylated N52 measured by biolayer interferometry (BLI). The experiments were performed two times and obtained consistent results, and representative data are shown.

Previous studies have demonstrated that the α-helix-promoting EAAAK sequence functions as a structural linker to increase the α-helicity of IPB29 (16). Here, we investigated whether EAAAK confers similar structural benefits to P43. Circular dichroism (CD) spectroscopy revealed that the intrinsic α-helical content of P43 (44%) was significantly higher than that of P37 (26%) (Fig. 7A). Upon incubation with the HR1-derived target-mimic peptide N52, both peptides formed stable α-helical complexes, exhibiting α-helical contents of 36% (P37/N52) and 47% (P43/N52). Notably, despite the increase in secondary structure, the thermal stability of the complexes was identical, with both P37/N52 and P43/N52 displaying a melting temperature (*T*_*m*_) of 79°C. This indicates that while the C-terminal addition of EAAAK substantially enhances the intrinsic α-helicity of P43, it does not improve the stability of the interaction with the target. Biolayer interferometry (BLI) further supported this observation, showing that P37 and P43 bound to N52 with comparable equilibrium dissociation constants (*K_D_*), measuring 5.62 × 10⁻⁸ M and 7.40 × 10⁻⁸ M, respectively (Fig. 7B). Additionally, both peptides exhibited high stability under various stress conditions, retaining full fusion-inhibitory activity following exposure to high temperatures, human serum, trypsin, or chymotrypsin, although they were susceptible to protease K digestion (Fig. S2A). Furthermore, neither peptide showed significant cytotoxicity at high concentrations across multiple cell lines (Fig. S2B). Using a truncated peptide P16 (Fig. 1), we also confirmed that EAAAK alone lacks antiviral activity (Fig. S2C).

### P43 peptide exhibits enhanced cellular uptake compared to P37

**Fig 8:**
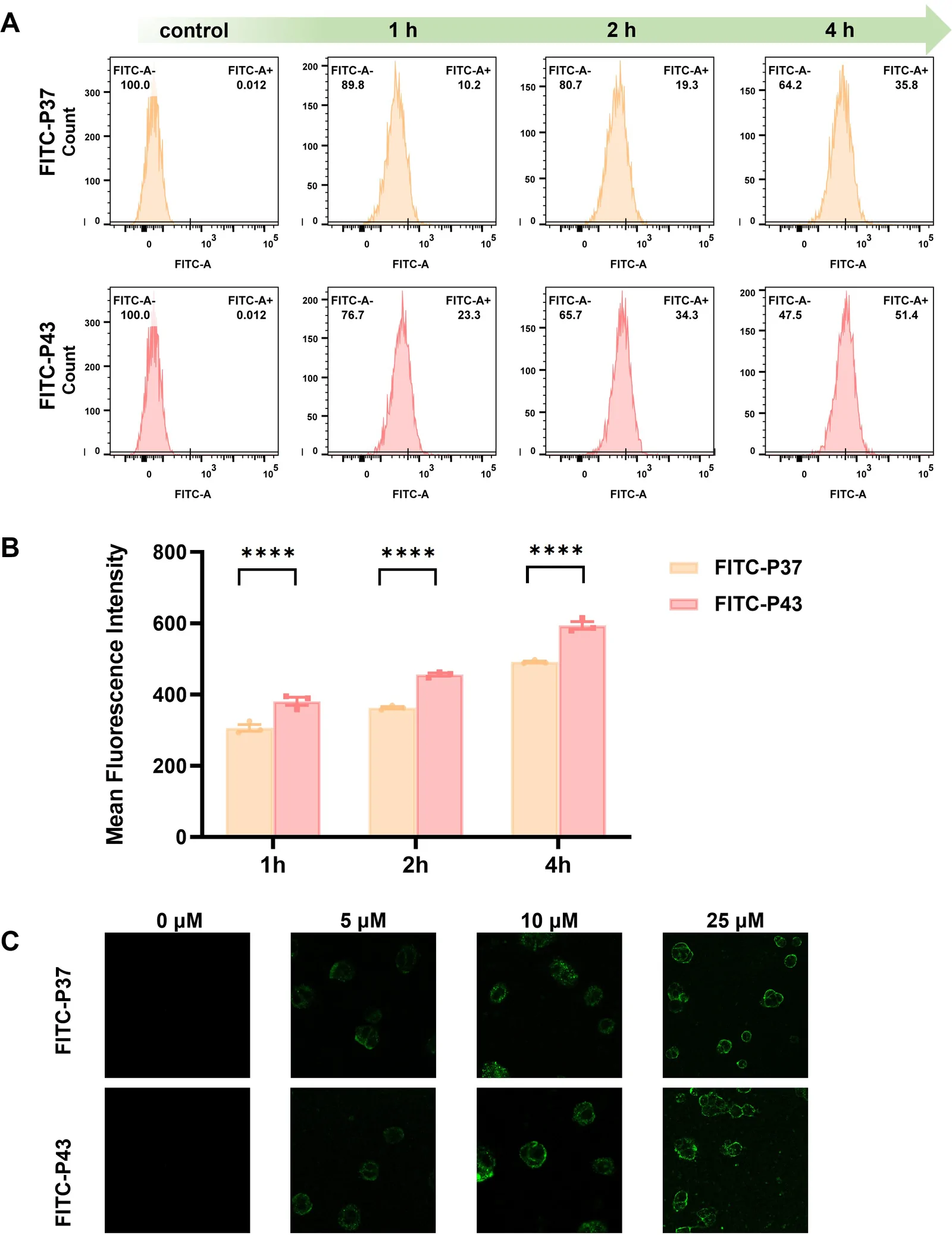
Cell membrane binding of P37 and P43. (**A**) Huh-7 cells were incubated with 25 µM of FITC-labeled peptides for the indicated times (0 min as control), and the proportion of FITC-positive cells was determined by flow cytometry. (**B**) Statistical analysis was performed using two-way analysis of variance, followed by Šidák’s multiple comparisons test: ns, not significant (*P* > 0.05); *P < 0.05; **P < 0.01; ***P < 0.001; ****P < 0.0001. (**C**) Huh-7 cells were incubated with 25 µM of FITC-labeled peptides at the indicated concentrations for 1 h, washed, and imaged by confocal microscopy.

**Fig 9:**
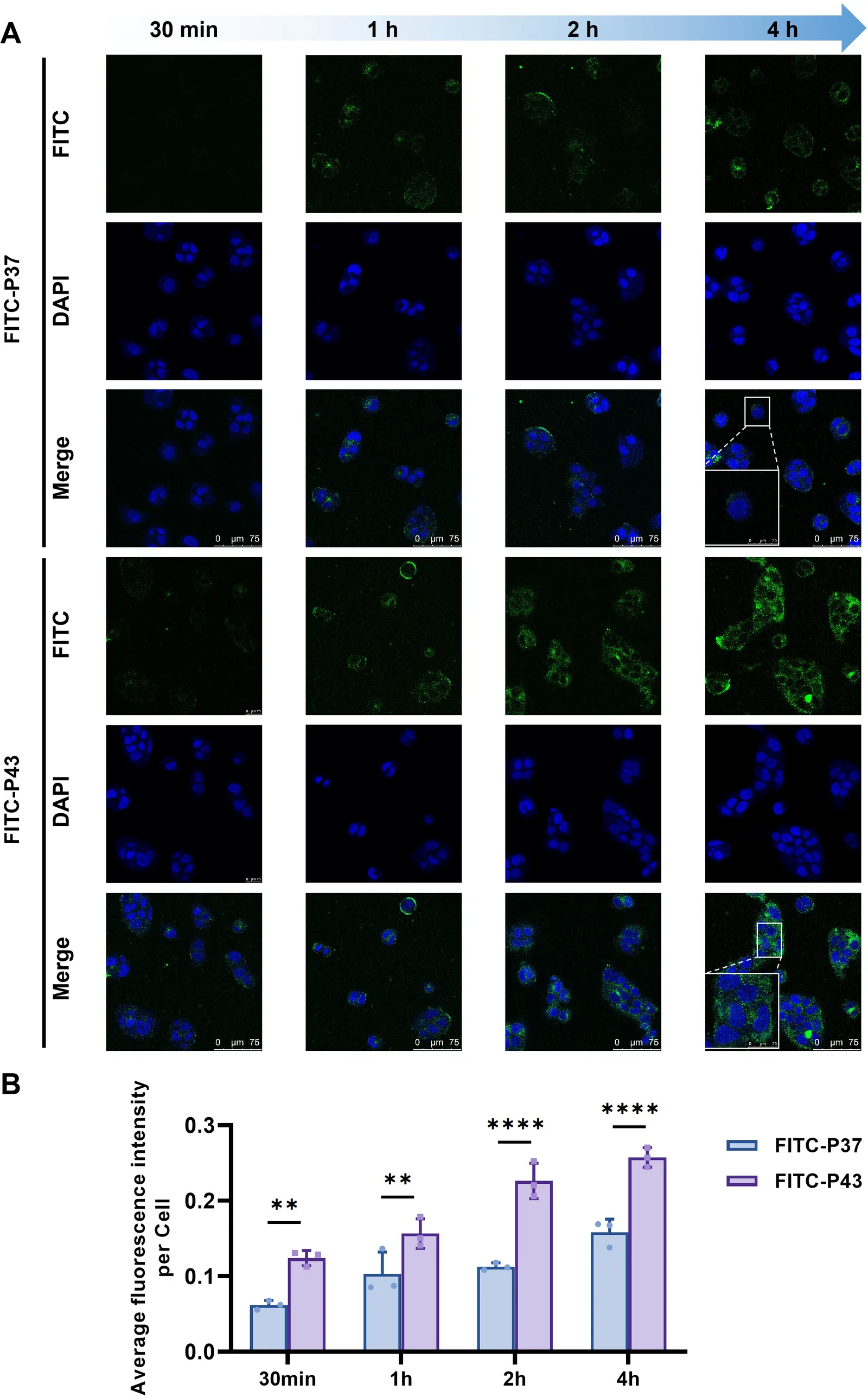
Cellular internalization of P37 and P43. (**A**) Huh-7 cells were incubated with 25 μM of FITC-labeled peptides for 30 min to 4 h, fixed, counterstained with DAPI, washed, and imaged by confocal microscopy. Images are representative of two independent experiments with similar results. In each independent experiment, images were acquired from three random fields. (**B**) Per-cell fluorescence intensity was measured per condition per field of view using Fiji (ImageJ), and error bars indicate SEM (*n* = 3). Statistical analysis was performed using two-way analysis of variance followed by Šidák’s multiple comparisons test: ns, not significant (*P* > 0.05); *P < 0.05; **P < 0.01; ***P < 0.001; ****P < 0.0001.

Given the superior antiviral activity of P43 in endocytosis-dependent infection models, we hypothesized that P43 might be internalized more efficiently than P37. To test this, we initially performed a cell-binding and washing assay. However, the peptides retained after washing showed no detectable inhibitory effect on either cell-cell fusion or pseudovirus infection (Fig. S3A and B), limiting the utility of this approach. We therefore synthesized FITC-labeled peptides (FITC-P37 and FITC-P43) for direct visualization. Flow cytometry analysis revealed that the cellular fluorescence levels increased over time for both peptides during a 1–4 h incubation period (Fig. 8A and B). Notably, while the mean fluorescence intensity of FITC-P43 was consistently higher than that of FITC-P37 at all tested time points, both peptides exhibited comparable proportions of FITC-positive cells, indicating similar initial membrane-binding capacities. Confocal microscopy confirmed that membrane binding was concentration-dependent for both peptides (Fig. 8C). Strikingly, time-course imaging revealed a distinct difference in intracellular distribution: FITC-P43 accumulated markedly in the perinuclear region over time, whereas FITC-P37 remained predominantly localized at the cell periphery with minimal internalization (Fig. 9A and B). In summary, the EAAAK sequence enhances the α-helical structure of the peptide and promotes its cellular internalization. This enables intracellular P43 to effectively inhibit endosomal membrane fusion, thereby accounting for its superior antiviral activity against viruses reliant on the endocytic entry pathways.

## DISCUSSION

Lipid conjugation has emerged as a viable strategy for designing peptide-based viral fusion inhibitors. The resulting lipopeptides generally exhibit substantially enhanced antiviral potency and *in vivo* stability compared to their unconjugated counterparts. Mechanistically, the lipid moiety promotes preferential interaction with viral and cellular membranes, thereby concentrating the inhibitor at the site of fusion. To facilitate this process, a flexible linker—such as a 4- or 8-unit polyethylene glycol (PEG_4_/PEG_8_) spacer or a glycine-serine repeat (GSGSG)—is typically inserted between the peptide and lipid to alleviate steric hindrance during membrane fusion. Consequently, rigid linkers have been considered unsuitable for this purpose. This rationale explains why PEG, GSGSG, or their combination have been predominantly used in developing lipopeptide fusion inhibitors against SARS-CoV-2, as seen in IPB02, IPB02V3, IPB24 (PEG_4_/PEG_8_) (12–14), EK1C4, P315V3, and SARS-HRC-PEG_4_-chol (GSGSG-PEG_4_ tandem) (19, 25, 26). In contrast, the α-helix-promoting sequence (EAAAK)*ₙ* has been widely employed in protein engineering as a rigid linker. It enhances the solubility and expression of recombinant fusion proteins by preventing misfolding and aggregation (27, 28), while also improving stability under physiological and storage conditions. Notably, we recently introduced the EAAAK sequence as a rigid linker in the SARS-CoV-2 fusion inhibitor IPB29. Contrary to conventional expectation, IPB29 demonstrated superior potency and breadth *in vitro* and *in vivo*, alongside favorable pharmacokinetic and safety profiles (9, 16, 20). In the present study, IPB29 again outperformed the lipopeptides IPB24 and IPB28—which share the same peptide sequence and cholesterol modification but use flexible PEG_4_ or GSGSG linkers—against emerging Omicron subvariants JN.1, KP.2, XDV.1, NB.1.8.1, and XFG. Collectively, these findings challenge the prevailing assumption that rigid linkers are unsuitable for lipopeptide fusion inhibitors and underscore the importance of elucidating the mechanism by which the EAAAK linker enhances antiviral activity.

SARS-CoV-2 enters host cells primarily through two distinct pathways mediated by its spike (S) glycoprotein: endosome-mediated membrane fusion and TMPRSS2-dependent surface membrane fusion (29, 30). The route adopted in a given cell line largely depends on the expression level of plasma membrane TMPRSS2 and the ability of the viral S protein to utilize this protease for entry (21, 22, 31, 32). Commonly used cell models such as 293T/ACE2 and parental Vero cells express relatively low levels of TMPRSS2, rendering them predominantly permissive to viral entry via endocytosis. In contrast, TMPRSS2-overexpressing Vero cells favor surface fusion, while Huh-7 and Caco-2 cells efficiently support both pathways (33, 34). From a virological perspective, earlier SARS-CoV-2 strains, including wild-type and early VOCs, are capable of entering via both routes, whereas Omicron subvariants exhibit a strong preference for the endosomal pathway (22, 23, 35–40). Previously, we reported that IPB29 exhibited enhanced inhibitory activity against diverse Omicron variants compared to earlier VOCs such as Alpha, Beta, and Delta (9, 16). Consistent with the entry tropism of Omicron, the subvariants JN.1, KP.2, and XDV.1 showed markedly reduced infectivity in Vero E6-TMPRSS2 cells compared to D614G and Delta. In this cell model, the three lipopeptides IPB24, IPB28, and IPB29 displayed similar potencies against D614G and Delta (Fig. 3). Importantly, we further demonstrated that IPB29 can effectively bind to and enter target cells, a property critical for its antiviral function (Fig. 4 and 5). Together, these findings not only indicate that the superior activity of IPB29—relative to IPB24 and IPB28—is linked to the endosomal entry pathway, but also underscore the intricate interplay among inhibitor, virus, and target cell, which warrants further detailed investigation.

The key finding of this study stems from the comparative analysis of two unconjugated template peptides, P43 and P37. Our results demonstrate that the addition of the EAAAK sequence substantially enhances the antiviral activity of the peptide in cell lines (Huh-7 and 293T/ACE2) that support both endosomal and surface fusion pathways (Fig. 6). In contrast, P43 and P37 exhibited comparable inhibitory potency in Vero E6-TMPRSS2 cells—which primarily utilize the surface fusion pathway—matching the behavior observed for their lipopeptide counterparts. Both peptides also showed similar efficacy in blocking S protein-mediated cell-cell fusion, a model of direct surface fusion. Mechanistically, circular dichroism spectroscopy and biolayer interferometry revealed that the EAAAK sequence markedly increases the α-helicity of the peptide, without altering its binding affinity for the HR1-derived target surrogate (Fig. 7). Flow cytometry and confocal microscopy further confirmed that P43 is internalized into target cells more efficiently than P37 (Fig. 8 and 9). Collectively, these data lead to two major conclusions: (i) the EAAAK sequence intrinsically boosts the antiviral activity of peptide inhibitors, functioning beyond its conventional role as a conjugation linker; and (ii) this enhancement is mediated primarily through improved cellular internalization, enabling more effective inhibition of viruses that rely on the endosomal entry pathway, while target binding affinity remains unchanged. However, it is worth noting that SARS-CoV-2 entry is multifaceted; since Omicron’s preference for endosomal entry may represent an *in vitro* artifact, the *in vivo* benefits of EAAAK may be limited in contexts where entry occurs strictly via plasma membrane fusion (21, 41–44).

In summary, this work uncovers a previously unrecognized function of the EAAAK sequence, shifting its paradigm from a passive structural linker to an active functional module that promotes intracellular delivery. This discovery provides a versatile strategy to enhance the potency of peptide-based inhibitors, particularly against viruses that rely on endocytic entry, such as SARS-CoV-2. More broadly, the EAAAK sequence may offer a generalizable platform for improving the cellular uptake and therapeutic efficacy of peptide drugs, with implications extending beyond antiviral development.

## MATERIALS AND METHODS

### Plasmids and cells

Plasmids encoding the SARS-CoV-2 spike (S) protein variants were generously provided by Linqi Zhang (Tsinghua University, Beijing, China) and Lu Lu (Fudan University, Shanghai, China). The DSP1-7 and DSP8-11 plasmids for the cell-cell fusion assay were kindly provided by Prof. Zene Matsuda (Institute of Medical Science, University of Tokyo, Tokyo, Japan). HEK293T, Vero E6, Caco-2, and Huh-7 cells were purchased from the American Type Culture Collection (ATCC; Rockville, MD, USA). Vero E6-TMPRSS2 and 293T/ACE2 cells, which stably express human TMPRSS2 or ACE2, respectively, were previously established in our laboratory. All cell lines were maintained in Dulbecco’s modified Eagle’s medium (DMEM) supplemented with 10% fetal bovine serum and 1% penicillin-streptomycin (complete medium). Vero E6-TMPRSS2 and 293T/ACE2 cells were cultured in complete medium further supplemented with 1 µg/mL or 6 µg/mL puromycin, respectively. Cells were incubated at 37°C in a humidified atmosphere containing 5% CO_2_.

### Synthesis of peptides and lipopeptides

The three SARS-CoV-2 fusion-inhibitory lipopeptides (IPB24, IPB28, and IPB29) used in this study were synthesized as described previously (16). The HR1-derived peptide N52 and the HR2-derived peptides P37 and P43 were synthesized by solid-phase peptide synthesis using Rink amide 4-methylbenzhydrylamine (MBHA) resin and standard 9-fluorenylmethoxycarbonyl (Fmoc) chemistry. All peptides were N-terminally acetylated on-resin prior to cleavage. Crude peptides were purified by reverse-phase high-performance liquid chromatography to >95% purity and verified by mass spectrometry.

### Single-cycle infection assay

The inhibitory activities of peptides and lipopeptides against SARS-CoV-2 variants were evaluated using a single-cycle infection assay as previously described (12). Briefly, pseudoviruses were generated by co-transfecting HEK293T cells with a plasmid encoding the SARS-CoV-2 spike protein and the Env-defective, luciferase-expressing HIV-1 backbone plasmid pNL4-3.luc.RE. After 6–8 h, the transfection medium was replaced with fresh DMEM. Virus-containing supernatants were harvested 48 h post-transfection, clarified by centrifugation, aliquoted, and stored at –80°C until use. For inhibition assays, pseudoviruses were pre-incubated with an equal volume of serially diluted peptide or lipopeptide (threefold dilution series) at 37°C for 30 min. Target cells (Huh-7, Caco-2, 293T/ACE2, Vero E6, or Vero E6-TMPRSS2) were then added at a density of 1.5 × 10⁴ cells per well. Following 48 h of incubation at 37°C, cells were lysed in reporter lysis buffer for 15 min. Luciferase activity was measured using luciferase assay reagents and a luminescence microplate reader (Promega, Madison, WI, USA).

### Cell-cell fusion assay

The inhibitory activity of peptides and lipopeptides on spike (S) protein-mediated cell-cell fusion was assessed using a dual-split-protein (DSP)-based assay, as previously described (12). Briefly, effector cells (293T cells) were seeded in a white clear-bottom 96-well plate at a density of 1.5 × 10⁵ cells/mL. Target cells (293T/ACE2) were cultured separately in a 10 cm dish. After overnight incubation at 37°C, 293T cells were co-transfected with a plasmid encoding the S protein and the DSP_1–7_ plasmid, while 293T/ACE2 cells were transfected with the DSP_8–11_ plasmid. Transfected cells were incubated for 24 h. Subsequently, effector cells were treated with serially threefold-diluted inhibitors for 1 h. Meanwhile, target cells were harvested, resuspended at 4 × 10⁵ cells/mL in pre-warmed culture medium containing 20 ng/mL EnduRen live-cell substrate (Promega), and incubated for 30 min. Thereafter, 2 × 10⁴ target cells per well were added to the effector cells, and the plate was centrifuged at 300 × *g* for 1 min to promote cell contact. Luciferase activity was measured using a luciferase assay system and a luminescence microplate reader (Promega).

### Assessment of cell-associated antiviral activity

To evaluate the membrane-binding capacity of peptide and lipopeptide inhibitors, Huh-7 or 293T/ACE2 cells were seeded in a 96-well plate at a density of 1.5 × 10⁴ cells per well. After incubation for 14–16 h at 37°C, the culture medium was removed, and diluted inhibitors were added to the cells and incubated for different durations (1, 2, or 4 h) at 37°C. The cells were then washed three times with phosphate-buffered saline (PBS, pH 7.2). The inhibitory activity of the cell-bound inhibitors remaining after washing was subsequently measured using the single-cycle infection assay or cell-cell fusion assay, as described in the previous sections.

### Confocal microscopy analysis

To visualize the binding and internalization of inhibitors, peptides were N-terminally labeled with fluorescein isothiocyanate (FITC). Vero E6 or Huh-7 cells were seeded in 20-mm glass-bottom dishes at 1.0 × 10⁵ cells per well and incubated for 14 h at 37°C under 5% CO_2_. The FITC-labeled inhibitors, diluted in culture medium, were then added and incubated for the indicated time periods. After incubation, cells were washed three times with PBS, fixed with 4% paraformaldehyde for 15 min, and counterstained with 4′,6-diamidino-2-phenylindole (DAPI) for 7 min. Fluorescence images were acquired using a laser scanning confocal microscope (Leica Microsystems, Wetzlar, Germany).

### Circular dichroism spectroscopy

The α-helical content and thermostability of peptide inhibitors and peptide complexes were analyzed by circular dichroism (CD) spectroscopy as described previously (12). Briefly, peptides were dissolved in PBS to a final concentration of 10 μM and incubated at 37°C for 30 min. CD spectra were acquired on a Jasco spectropolarimeter (models J-815 and J-1700) with a bandwidth of 1 nm and a step resolution of 1 nm, scanning from 195 to 270 nm at room temperature. Spectra were corrected by subtracting the solvent blank. The α-helical content was calculated from the mean residue ellipticity ([θ]) at 222 nm, using the equation: % α-helix = ([θ]₂₂₂ nm/–33,000) × 100, where –33,000 deg·cm²·dmol⁻¹ corresponds to a fully helical structure. For thermal denaturation studies, the ellipticity at 222 nm was monitored as the temperature increased from 20°C to 98°C at a rate of 2°C/min. The melting temperature (*T*_*m*_) was defined as the midpoint of the unfolding transition.

### Biolayer interferometry

The binding affinity between peptide inhibitors and an HR1-derived target mimic peptide (N52) was determined using biolayer interferometry (BLI) as previously described (45). Briefly, biotinylated N52 (biotin-N52) was synthesized by N-terminal biotin labeling. Biotin-N52 was diluted to 5 µg/mL in PBS and immobilized onto streptavidin biosensors (Sartorius, Göttingen, Germany) until a response shift of approximately 4 nm was achieved. Peptide inhibitors were serially diluted in PBS-T buffer (PBS containing 0.2% Tween-20). Binding kinetics were measured using an association phase of 60 s, followed by a dissociation phase of 180 s in PBS-T alone.

### Flow cytometry analysis

The membrane binding capacity of P37 and P43 peptides was assessed by flow cytometry. Huh-7 cells were seeded in six-well plates at 3.0 × 10⁵ cells per well and incubated for 14 h at 37°C under 5% CO_2_. The FITC-labeled inhibitors, diluted in culture medium to 25 μM, were then added and incubated at 37°C for the indicated time periods. Cells were then washed three times with PBS to remove unbound fluorescent dye and digested them from the well plate. Cells were then centrifuged at 2,000 rpm for 2 min, washed three times with flow cytometry buffer (PBS + 1% bovine serum albumin), and filtered through a cell strainer. Fluorescence was measured on a FACSCanto II flow cytometer (Becton Dickinson, Mountain View, CA, USA).

### Metabolic stability assay

The metabolic stability of P37 and P43 was evaluated by monitoring their antiviral activity over time after exposure to various conditions, using the D614G spike protein-based cell–cell fusion assay described above. For thermal stability, peptides were stored at 25°C or 37°C for up to 63 days and tested at regular intervals. For serum stability, peptides were incubated with 20% fresh human serum at 37°C for 15, 30, 60, 120, 180, and 240 min. Protease stability was assessed by incubating peptides with trypsin, chymotrypsin, or proteinase K (each at a final concentration of 2 mg/mL) at 37°C for the same time points.

### Cytotoxicity assay

The *in vitro* cytotoxicity of P37 and P43 was evaluated using a Cell-Counting Kit-8 (CCK-8) assay. Briefly, 293T/ACE2, Huh-7, or Vero E6-TMPRSS2 cells were seeded in 96-well tissue culture plates at a density of 1–1.5 × 10⁴ cells per well. Peptides diluted to the indicated concentrations were added in 50 μL volumes. After 48 h of incubation at 37°C, 20 μL of CCK-8 solution was added to each well, followed by an additional 30-min incubation at 37°C. Absorbance at 450 nm was measured using a Multiskan MK3 microplate reader (Thermo Fisher Scientific, Waltham, MA, USA). Cell viability was expressed as a percentage relative to untreated controls.

### Statistical analysis

Data were analyzed using GraphPad Prism 8 (GraphPad Software Inc.). Significance levels are denoted as follows: ns, not significant; *P < 0.05, **P < 0.01, ***P < 0.001, and ****P < 0.0001. A *P*-value of less than 0.05 was considered statistically significant.

## Data Availability

Data will be made available on request.

## SUPPLEMENTAL MATERIAL

Fig. S1 jvi.01067-26-s0001.tif Cellular binding capacity of lipopeptide-based fusion inhibitors.

Fig. S2 jvi.01067-26-s0002.tif Metabolic stability and *in vitro* cytotoxicity of P37 and P43 peptides.

Fig. S3 jvi.01067-26-s0003.tif Inhibitory activity of cell-associated P37 and P43 peptides.

Supplemental legends jvi.01067-26-s0004.docx Legends for Fig. S1 to S3.

ASM does not own the copyrights to Supplemental Material that may be linked to, or accessed through, an article. The authors have granted ASM a non-exclusive, world-wide license to publish the Supplemental Material files. Please contact the corresponding author directly for reuse.
