# Supplementary material for: The α-helix-facilitating amino acid sequence EAAAK dramatically increases the antiviral activity of SARS-CoV-2 fusion-inhibitory peptides via the endosome fusion pathway
Source: J Virol. 2026 Sep 8;100(10):e01067-26. doi: 10.1128/jvi.01067-26 (PMC13712796; doi:10.1128/jvi.01067-26)
Supplement: Supplementary file 1 [file jvi.01067-26-s0004.docx]

**Supplemental Materials**

**Supplemental legends:**

**Figure S1.** **Cellular binding capacity of lipopeptide-based fusion inhibitors.**

**(A)** Sustained antiviral activity of cell-bound IPB29 and control inhibitors (Camostat, the fusion-inhibitory peptide P39, and the RBD-targeting fusion protein LCB1T-Fc) against SARS-CoV-2 pseudoviruses. Inhibitors were pre-incubated with Huh‑7 cells for 1 h, washed thoroughly, and their residual inhibitory activity against D614G was assessed using a pseudovirus-based single-cycle infection assay. Data are presented as mean ± SD from three independent experiments, each performed in triplicate. **(B)** Membrane binding capacity of lipopeptides

**Figure S2. Metabolic stability and in vitro cytotoxicity of P37 and P43 peptides.**

**(A)** Stability profiles of P37 and P43. Peptides were exposed to elevated temperature, human serum, or proteases (trypsin, chymotrypsin, or proteinase K). Their residual antiviral activity over time was assessed by a DSP-based cell–cell fusion assay. For proteinase K‑treated samples, IC_50_ could not be determined due to near‑complete loss of activity; percent inhibition at the starting concentration (417 nM) is shown instead. **(B)** Cytotoxicity of P37 and P43. Cell viability of Huh‑7, 293T/ACE2, and Vero E6-TMPRSS2 cells treated with increasing concentrations of peptides was evaluated using a Cell‑Counting Kit‑8 (CCK‑8) assay. Experiments were done twice and one representative result was shown. Error bars indicate SEM of technical triplicates. **(C)** Inhibitory activity of truncated peptide P16 determined by a pseudovirus-based single-cycle infection assay (left panel) or DSP-based cell–cell fusion assay (right panel). Data are presented as mean ± SD from three independent experiments, each performed in triplicate.

**Figure S3.** **Inhibitory activity of cell-associated P37 and P43 peptides.**

**(A)** Inhibitory activity of cell-bound P37 and P43 against D614G spike-mediated cell‑cell fusion. Peptides were pre‑incubated with 293T/ACE2 cells for 1 h, thoroughly washed, and residual activity against D614G spike protein was measured using a dual DSP‑based cell fusion assay. **(B)** Inhibitory activity of cell-bound P37 and P43 against the BA.1 variant. Peptides were pre‑incubated with Huh‑7 cells for 4 h, washed thoroughly, and remaining inhibition of BA.1 pseudovirus infection was assessed by single‑cycle infection assay. Data are presented as mean ± SD from three independent experiments.
